# Determination of optimum oven cooking procedures for lean beef products

**DOI:** 10.1002/fsn3.229

**Published:** 2015-04-24

**Authors:** Argenis Rodas‐González, Ivy L. Larsen, Bethany Uttaro, Manuel Juárez, Joyce Parslow, Jennifer L. Aalhus

**Affiliations:** ^1^ Department of Animal ScienceFaculty of Agricultural & Food SciencesUniversity of Manitoba,201 Animal Science/Entomology Building ‐ 12 Dafoe RoadWinnipegManitoba R3T 2N2Canada; ^2^ Agriculture and Agri‐Food CanadaLacombe Research Centre,6000 C & E TrailLacombeAlbertaT4L 1W1Canada; ^3^ Canada Beef Inc,4‐101, 2000 Argentina RoadMississaugaOntarioL5N 1W1Canada

**Keywords:** Beef, cookery method, digital imaging, roasting, searing, tenderness

## Abstract

In order to determine optimum oven cooking procedures for lean beef, the effects of searing at 232 or 260°C for 0, 10, 20 or 30 min, and roasting at 160 or 135°C on semimembranosus (SM) and *longissimus lumborum* (LL) muscles were evaluated. In addition, the optimum determined cooking method (oven‐seared for 10 min at 232°C and roasted at 135°C) was applied to SM roasts varying in weight from 0.5 to 2.5 kg. Mainly, SM muscles seared for 0 or 10 min at 232°C followed by roast at 135°C had lower cooking loss, higher external browning color, more uniform internal color, and were more tender and flavorful (*P *<* *0.05). Roast weights ≥1 kg had lesser cooking loss, more uniform internal color and tender compared to 0.5 kg (*P *<* *0.05). Consequently, roasting at low temperature without searing is the recommended oven cooking procedure; with best response from muscle roast weight ≥1 kg.

## Introduction

The Beef Information Centre (BIC 2004) indicated that there are five different cooking instructions for beef roasts: Pot Roast, Rotisserie Roast, Quick Roast, Oven Roast, and Premium Oven Roasts. However, a marketing research study (BIC 2004) indicated that consumers cannot clearly differentiate between them. At the same time, consumers are expecting a “premium” eating experience without complicated cooking procedures (Jeremiah and Gibson 1999).

A generally accepted method of cooking beef roast is to dry roast at a constant temperature throughout the cooking time, particularly for cuts of beef with low connective tissue content; however, most muscles with high connective tissue content (i.e., semimembranosus; SM) are thought to require moist heat cookery, such as braising (Cheng and Sun 1992). Nevertheless, some researchers have reported that adding water during the cooking process produces negative effects on palatability attributes and cooking loss (McDowell et al. 1986; Jeremiah and Gibson 1999; Bejerholm and Aaslyng 1969). Instead of moist heat cookery, low temperature dry heat cookery has been recommended (Jeremiah and Gibson 1999). Moist heat cookery methods could be improved by applying a prebrowning or searing (high initial temperature). Searing the meat creates a surface barrier of denatured proteins which may reduce the moisture loss, odor component loss (i.e., soluble proteins and fat) and facilitate the Maillard reaction (Whitfield and Mottram 2000; Aalhus et al. 2007), developing an appropriate roasted flavor and aroma (Jeremiah and Gibson 1999).

In the food service industry, cooking methods have developed which combine searing with low temperature roasting, yielding tender meat despite high contents of connective tissue. The slow rate of heat transfer is thought to hold temperatures within the 50–60°C range for a longer period of time resulting in more softening of collagen and a slower coagulation of the myofibrillar proteins, resulting in less loss of moisture (Bramblett et al. 1981; Bouton and Harris 2005).

To determine if this cooking approach could be adapted to a simple method for home cookery, the effects of high temperature oven searing and subsequent low temperature roasting on palatability and color attributes was evaluated. Searing at different temperatures (232 or 260°C) and times (0, 10, 20 or 30 min) combined with roasting temperatures (160 or 135°C) were tested on SM inside round roasts representative of a high connective tissue standard oven roast and LL strip loin roasts representative of a low connective tissue premium oven roasts. Further testing was carried out in order to determine if the optimum roasting cooking procedure determined previously, was equally applicable over a range of roast weights (0.5, 1, 2 or 2.5 kg).

## Materials and Methods

### To establish optimum searing conditions (temperature and time) and roasting temperatures for semimembranosus and longissimus lumborum roasts

A total of 48 inside round subprimals (approximate weight 4.5 kg) from graded Y1 AA (Canada Gazette 1959), cattle were obtained from a commercial slaughter plant over two subsequent weeks and shipped to the Agriculture and Agri‐Food Research Centre (Lacombe AB, Canada). The subprimals were aged at 2°C in vacuum packages for 14 ± 1 day from the date of slaughter. Twelve inside rounds were assigned to each searing time (0, 10, 20, or 30 min). Two to four days prior to cooking, the subprimals were removed from their packaging and *semimembranosus* (SM) muscles were dissected.

The SM muscles were trimmed to a square shape and two steaks (2.5 cm thick) from the proximal portion of the muscles were removed, one for determination of initial shear force and one for determination of initial moisture and fat content. The remainder of the SM muscle was divided into two equal weight roasts (1.28 ± 0.06 kg). Controlling for location within muscle, the 24 roasts within each searing time were equally distributed to each searing (232 or 260°C) and roasting temperature (160 or 135°C) combination (*n* = 6). Roasts were labeled, placed into polyethylene bags, and stored in a refrigerator (Norlake Scientific Model NSPR803WW/8, Nor‐Lake Inc. Hudson, WI) at 4°C until cooked.

For the *longissimus lumborum* (LL), 48 strip‐loins were shipped to the AAFC Research Centre from the same packing plant as for the inside rounds. Processing of strip‐loins was conducted in the same manner as described above (i.e., dissection, aging, subset, obtaining steaks, etc.). LL portions were trimmed to a final weight of 1.28 ± 0.06 kg and assigned to searing and roasting treatments in the same manner.

### Cooking procedure

Before cooking, roasts from both the LL and SM were weighed (Ohaus Balance GT4100 Fisher Scientific, Mississauga, ON, Canada) and placed in a labeled oval roasting pan (28 × 18 × 7 cm) on a wire rack. Type T thermocouple probes (Wika Instruments, Edmonton, AB, Canada) with a fiberglass stainless steel covering and 20 gauge lead wire, rated for 480°C, were inserted in the center of each roast. Muscles were tempered at room temperature (25°C) until the internal temperature of the muscle reached 10 ± 2°C; then muscle portions were placed into conventional electric ovens preheated to 232 or 260°C (Frigidaire Gallery Series, Model PGLEF385ES2, Electrolux Canada Corp, ON, Canada) two roasts per oven, evenly spaced on the lowest oven rack (5 cm from the bottom). The cooking time and temperature rise were monitored every 10 sec (Hewlett Packard HP34970A Data Logger [Hewlett Packard Co., Boise, ID). Roasts were seared at their assigned temperature (232 or 260°C) for their specific searing time (0, 10, 20 or 30 min). The ovens were then turned down to the required roasting temperature (160 or 135°C) and roasts were cooked until final internal temperature of 68°C. A temperature probe was mounted in both ovens to monitor and record oven temperatures at 10 sec intervals.

Upon reaching the target internal temperature, the roasted muscle and wire cooking rack were removed from the roasting pan and placed on a preweighed tray to collect drip during cooling. The cooling roast was immediately covered with a piece of high strength alloy aluminum foil, with the shiny side out. The thermocouple remained inserted in the roasted muscle until maximum rise was attained and the temperature proceeded to drop. After the temperature drop, the thermocouple was removed and the roast was left to stand on the rack of the preweighed tray and remained covered with the foil before weighing (20 min from time roast was removed from oven). The weight of the cooked roasted muscle and cooling drip were weighed separately and recorded.

The degree of external browning of the roasted muscle was rated by technical staff. Then, the roasted muscle was halved across the long axis of the fibers. One half was used for sensory analysis and the second for subjective and instrumental color evaluation (utilizing image analyses) of internal degree of doneness, shear force analysis and residual moisture, and fat content in the cooked product.

### Moisture and fat content

Raw and roasted muscle samples (~150 g) were cut into small pieces and ground for 10 to 15 sec (Robot Coupe Blixir BX3, Robot Coupe USA Inc., Ridgeland, MS). This homogenate was subsampled for determination of moisture content, crude intramuscular fat extracted with petroleum ether, and crude protein by nitrogen determination (Aldai et al. 2004).

### Subjective and instrumental evaluation of color

Subjective color assessments were performed by two trained panellists not involved in the cooking process. To rate the external surface roast color, a 6‐point descriptive scale was used (1 = Slight brown; 2 = Light brown; 3 = Red brown; 4 = Dark brown; 5 = Excessive brown; 6 = Charred and almost burnt). For internal degree of doneness, a 7‐point descriptive scale was used (1 = Very rare; 2 = Rare; 3 = Medium rare; 4 = Medium; 5 = Medium well done; 6 = Well done; 7 = Very well done). The scale was a modification of the 6‐point scale used by AMSA (2010) with the addition of a “medium well done” category.

To evaluate the homogeneity of cooked roast color a 1.9 cm‐thick steak was obtained from the center portion of the roasted muscle for image analyses. Within approximately a minute of exposure to the air, images of each surface were digitally captured (2272 × 1704 pixels; 72 dpi) with a digital camera (Canon PowerShot A80, Canon USA, Inc., Lake Success, NY) fitted with a polarizing filter adjusted 90° to the orientation of polarizing filters on the two GE 100W Reveal lights illuminating the sample from 45° to the horizontal. A custom white balance was set from a Kodak 18% grey card. Image J (v 1.32j; available at http://rsb.info.nih.gov/ij; developed by Wayne Rasband, National Institutes of Health, Bethesda, MD) was used to select the area of interest, divide the 256 × 256 × 256 RGB color space into 16 × 16 × 16 bins for a total of 4096 bins, and record the number of pixels within each bin. Color uniformity was understood to be inversely related to the number of bins used.

### Shear force analysis

For initial Warner–Bratzler shear force (WBSF), steak samples were cooked on an electric grill to a final temperature of 71°C (AMSA 2010). After cooking, steaks were held in a cooler for a 24 h period and then three cores from fixed locations (1.9 cm in diameter) per steak were removed parallel to the fiber direction and sheared (Instron 4301 Material Testing System equipped with a Warner–Bratzler cell and Series 9 Software, Instron Canada, Burlington, ON, Canada) as described by Aalhus et al. (2004). Peak shear force was recorded as the average of three 1.9 cm diameter cores). From the roasted muscle, a 2.5 cm thick steak was obtained and chilled overnight for assessment of objective shear as described above.

### Sensory evaluation of palatability attributes

Six 3 mm thick slices (Globe Meat Slicer, Model 500 Gravity Feed, Globe Slicing Machine Company Inc, Stamford, CN) were removed from the halved roasted muscle for taste panel samples. All six slices were weighed as a group to determine juice losses during slicing. Percent of juice loss was calculated at the one‐gram level using the following formula: (whole cooked roasted muscle before slicing, total weight of six slices)/(whole cooked roasted muscle before slicing) × 100.

Immediately following weighing, the first slice was discarded and the remaining five slices were placed on a clean‐cutting board and four slices were chosen for size uniformity. Each of the four slices was trimmed to 7.6 cm wide and 6.3 cm long, and then cut into two similar lengthwise pieces from the center out with the top external browned surface left on, for a total of eight pieces each per roast. Further, the eight samples were rolled, secured with a toothpick, and placed into two prewarmed 250 mL glass jars in a 68°C circulating hot water bath (Lindberg/Blue Model WB1120A‐1, Kendro Laboratory Products, Asheville, NC) until all four roast samples had been prepared. Four samples were served to each panellist. Panellists were seated in well‐ventilated, partitioned booths, under red lighting. Rolled samples were served warm, without seasoning, accompanied by a glass of deionized water and unsalted crackers to cleanse their palate after tasting each sample (Larmond 1970b).

Panellists were instructed to bite across the roll of the sample on the side farthest from the toothpick, approximately 1 cm from the end. Attribute ratings were electronically collected (Compusense Inc., Guelph, ON, Canada) using an 8‐point descriptive scale for initial and overall tenderness (8 = extremely tender; 1 = extremely tough), juiciness (8 = extremely juicy; 1 = extremely dry), beef flavor and browning intensity (8 = extremely intense; 1 = extremely bland/none) and amount of connective tissue (8 = none detected; 1 = extremely abundant). Comments were electronically collected about the flavor or texture, if noted.

### To determine if the optimum roasting conditions were applicable over a wide range in muscle weights

Right and left SM were fabricated from six carcasses (Grade AA; Canada Gazette 1959) at 24 h. Muscles were labeled, vacuum packaged, and stored at 2°C for 14 day. After aging, SM were removed from their packaging, trimmed to a square shape and two steaks (2.5 cm thick) were removed from the proximal portion, one for the determination of initial shear force, and one for moisture and fat content as described previously. Each SM, alternating between right and left sides, was further subdivided into two (either 0.5 and 2.5 kg roast, or a 1.0 and 2.0 kg roast) for a total of four roasts per animal. Roasts were labeled, placed into polyethylene bags and stored at 2°C until cooking. All four roasts from the same animal were cooked in the same oven under the same conditions (e.g., oven seared for 10 min at 232°C, oven temperature dropped to 135°C, and roasted to a final end point temperature of 68°C). Laboratory analyses were as described above, except no taste panel data were collected.

### Statistical analysis

Data collected were analyzed using the MIXED procedure of SAS (Cary, NC) version 9.2 (SAS 1997). To analyze the effect of searing time, searing temperature, and roasting temperature, a completely balanced design with split‐plot arrangement was used. Searing time (0, 10, 20, and 30 min) and searing temperature (232 or 260°C) were in the whole‐plot while roasting temperature (135 or 160°C) was in the subplot. Thus, searing time, searing temperature and roasting temperature, and their interactions were all considered to be fixed. Random variables included replication nested within searing time, searing temperature, and roasting temperature. Data across muscles (SM or LL) were analyzed separately. To examine the effects of different roast sizes (0.5, 1, 2 or 2.5 kg), the weight group was considered as a fixed effect. The random variable included animal, as well as roast weight group nested within a side.

For all statistical models, least squares means were separated (*F* test, *P *<* *0.05) by using least significant differences generated by the PDIFF option. The degrees of freedom in the denominator were adjusted using the Kenward–Roger procedure. Initial shear force and fat content were included in the models as covariates.

## Results

### Establishing optimum searing conditions (temperature and time) and roasting temperatures for semimembranosus and *longissimus lumborum* roasts

#### Cooking traits

**Figure 1:**
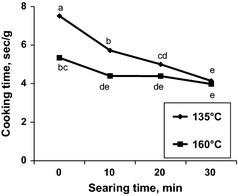
Cooking time required to reach internal temperature of 68°C in *Longissimus lumborum* roasts seared for 0, 10, 20, 30 min and roasted at 135 and 160°C. SEM = 0.24; *P *=* *0.005.

In the SM standard oven roasts, searing for 0 min required more time to reach 68°C, followed by searing 10 and 20 min and finally searing for 30 min (*P *<* *0.001; Table 1); as well, roasting at 135°C required more time than roasting at 160°C (*P *=* *0.01). In contrast, for the LL premium roasts, the interaction for searing time × roasting temperature (Figure 1; *P *=* *0.005) indicated that cooked LL samples seared for 0 min and roasted at 135°C required more time to reach 68°C than the other treatments (*P *<* *0.05). However, as searing time increased, differences between roasting temperatures disappeared and cooking time was reduced; the shortest cooking time was recorded for samples seared for 30 min and roasted either at 135 or 160°C. In addition, the main effect of searing temperature affected the cooking times (*P *=* *0.02), where samples seared at 232°C needed more time to reach the final internal temperature of 68°C than samples seared at 260°C.

**Table 1:** Cooking traits and postcooking proximate analysis of beef s*emimembranosus* and *longissimus lumborum* roasts cooked at different searing time, searing temperature and roasting temperature.

Variable	STI, min				STE, °C		RTE, °C		SEM	*P*‐value		
	0	10	20	30	232	260	135	160		STI	STE	RTE
*Semimembranosus*												
Cooking time, sec/g	6.97^a^	6.38^b^	6.30^b^	5.50^c^	6.43	6.14	7.14	5.44	0.126	<0.001	0.055	<0.001
Cooking loss, %	24.9^b^	26.6^a^	24.9^b^	25.8^ab^	25.4	25.7	25.0	26.0	0.330	0.019	0.368	0.015
Moisture loss during slicing, %	4.29^a^	2.32^c^	3.02^bc^	3.19^b^	3.26	3.15	3.13	3.28	0.213	<0.001	0.679	0.561
Moisture, %	64.9^a^	63.5^b^	64.0^ab^	64.6^a^	64.6	64.0	64.3	64.3	0.263	0.032	0.071	0.992
Fat, %	3.08^c^	3.74^ab^	3.83^a^	3.10^bc^	3.32	3.56	3.51	3.36	0.166	0.019	0.237	0.431
Protein, %	31.2	31.9	31.5	31.7	31.4	31.7	31.5	31.7	0.186	0.235	0.138	0.398
*Longissimus lumborum*												
Cooking time, sec/g	6.43^a^	5.06^b^	4.70^b^	4.06^c^	5.25	4.87	5.59	4.53	0.136	<0.001	0.027	<0.001
Cooking loss, %	24.9^b^	24.8^b^	24.7^b^	27.1^a^	24.9	25.8	25.2	25.5	0.296	<0.001	0.011	0.486
Moisture loss during slicing, %	2.90^a^	2.85^a^	2.36^b^	2.34^b^	2.55	2.62	2.43	2.74	0.116	0.003	0.656	0.038
Moisture, %	64.8^ab^	65.2^a^	64.5^b^	63.8^c^	64.8	64.4	64.6	64.5	0.193	0.001	0.099	0.691
Fat, %	3.49	3.97	3.92	4.12	3.84	3.91	3.88	3.87	0.180	0.243	0.744	0.973
Protein, %	30.6^b^	30.4^b^	30.8^ab^	31.2^a^	30.6	30.9	30.7	30.8	0.136	0.009	0.075	0.595

STI, Searing time; STE, Searing temperature; RTE, Roasting temperature; SEM, Pooled standard error of least square means.

^a,b,c^Least squares means within a row lacking a common superscript letter differ (*P *<* *0.05).

In the SM standard oven roasts, searing for 0 and 20 min (Table 1) resulted in lower cooking loss (*P *<* *0.05) than searing for 10 min, while searing for 30 min resulted in intermediate values. It was expected that cooking loss might decrease as searing time increased due to denaturation of the surface proteins reducing water loss, but the results were inconsistent. At the same time, roasting at 135°C had less cooking loss than roasting at 160°C (*P *=* *0.01). For the LL premium oven roasts (Table 1), searing for 0, 10 or 20 min resulted in lower cooking loss than searing for 30 min (*P *<* *0.05). As well, searing temperatures of 232°C had lower cooking loss compared to searing at 260°C (*P *=* *0.01).

For moisture loss during slicing of SM roasts (Table 1), searing for 0 min (no searing) resulted in greater moisture loss than the other searing times whereas searing for 10 min resulted in the least moisture loss (*P < *0.05). For LL roasts, searing for 0 or 10 min resulted in greater moisture loss during slicing than searing for 20 or 30 min (*P *<* *0.05). Furthermore, moisture loss during slicing was higher in samples roasted at 160°C than those roasted at 135°C (*P *=* *0.03). Higher moisture loss during slicing was expected on samples with lower cooking losses (more water retention) as they would express more water when sliced.

#### Proximate analysis

For SM samples taken after roasting (Table 1), as expected, searing for 10 min, which had higher cooking losses than the others searing times (*P *<* *0.05), resulted in less moisture content than searing at 0 and 30 min. As well, searing for 0 min resulted in lower fat content than searing for 10 and 30 min (*P *<* *0.05). No difference in protein content was detected (*P *>* *0.05).

For LL roasted samples (Table 1), there was a significant effect of searing time on moisture (*P *=* *0.001) and protein content (*P *=* *0.009). Searing for 30 min resulted in lower moisture and higher protein content than the other searing time treatments. Fat content was not affected by any of treatments (*P *>* *0.05).

#### Color evaluation

In the SM standard oven roast, external browning was affected primarily by roasting temperature (*P *<* *0.001). To achieve the same degree of doneness, the longer roasting times at 135°C produced higher browning scores than shorter roasting times at 160°C (Table 2). On the contrary, searing time had an effect on the visual perception of internal degree of doneness (*P *=* *0.04) on the roast cross‐section, although not on external browning (*P *>* *0.05). Thus, searing for 0 and 10 min, which resulted in longer cook times, caused higher visual perceptions of the internal degree of doneness, being scored as “medium well done”. In contrast searing for 30 min, resulted in shorter cook times, and caused lower visual perceptions of the internal doneness, being scored as “medium”. Searing for 20 min resulted in intermediate values. Results from analysis of images of the entire internal surface area of the cooked SM oven roasts to determine uniformity of doneness indicated that although the total number of bins was uniform across treatments, the number of bins required to hold 75% of the pixels was affected by searing time (*P *=* *0.03); fewer color bins were used, indicating a more uniform color, when searing 0 to 20 min than when searing for 30 min.

**Table 2:** Color evaluation of panellists and digital imaging of beef s*emimembranosus* and *longissimus lumborum* roasts cooked at different searing time, searing temperature and roasting temperature.

Variable	STI, min				STE, °C		RTE, °C		SEM	*P*‐value		
	0	10	20	30	232	260	135	160		STI	STE	RTE
*Semimembranosus*												
External Browning^a^	2.25	2.30	2.53	2.20	2.45	2.19	2.80	1.83	0.153	0.589	0.148	<0.001
Internal Doneness^b^	5.02^a^	4.98^a^	4.66^ab^	4.47^b^	4.77	4.79	4.73	4.83	0.116	0.040	0.877	0.444
Total Bins used	143.9	140.6	141.3	150.6	141.15	147.02	146.31	141.85	3.216	0.359	0.129	0.248
Bins holding 75% of pixel	6.40^b^	6.23^b^	6.65^b^	7.47^a^	6.69	6.69	6.92	6.46	0.226	0.031	1.000	0.094
*Longissimus lumborum*												
External Browning^a^	2.78^a^	2.43^a^	1.65^b^	1.46^b^	2.19	1.97	2.29	1.87	0.133	<0.001	0.193	0.011
Internal Doneness^b^	5.00^ab^	4.75^b^	5.04^a^	5.16^a^	4.98	5.00	4.98	5.00	0.076	0.020	0.822	0.822
Total Bins used	145.2^b^	161.3^a^	157.5^ab^	163.5^a^	158.64	155.06	158.54	155.16	3.600	0.032	0.430	0.457
Bins holding 75% of pixel	8.94	9.54	9.32	9.18	9.37	9.11	9.41	9.08	0.346	0.798	0.554	0.458

STI, Searing time; STE, Searing temperature; RTE, Roasting temperature; SEM, Pooled standard error of least square means.

^a,b,c^Least squares means within a row lacking a common superscript letter differ (P < 0.05).

a Degree of external browning 1 = a slight browning to 6 = charred/almost burnt.

b Degree of visual perception of doneness 1 = very rare to 6 = very well done.

In contrast to the SM standard oven roast, in the LL premium roasts (Table 2) searing for 0 and 10 min, resulting in longer cook times, yielded higher external browning color scores compared to the shorter cook times associated with searing for 20 and 30 min (“light brown” vs. “slight brown). However, searing for 10 min resulted in the perceived visual internal degree of doneness being lower than the other searing times (“medium” vs. “medium well done”). In the LL roasting at 135°C also produced higher external browning scores than roasting at 160°C without affecting visually perceived degree of doneness. Results from analysis of digital images of LL cooked roasts cross‐section showed that the total bins used was affected by searing time (*P = *0.03; Table 2); searing for 0 min used fewer bins than other searing times, indicating a more uniform color across the internal surface.

#### Shear force and sensory evaluation

The WBSF and palatability attributes of beef SM and LL roasts cooked at different searing time, searing temperature and roasting temperature are presented in Table 3. Despite significant differences in cooking and color parameters associated with searing time, there were few differences in the SM roast palatability characteristics. A tendency for higher scores for beef flavor intensity (*P *=* *0.07) and browning flavor intensity (*P *=* *0.06) associated with the longer cook times arising from 0 min of searing was observed. Additionally, searing time x searing temperature interaction on the browning flavor score was observed (*P *=* *0.02; data not shown). Searing until 20 min either at 232 or 260°C did not produce changes on browning flavor score, but for 30 min a higher browning flavor rating (“moderately bland”) was assigned at 260°C than at 232°C (“very bland”). Roasting the SM at 135°C resulted in higher scores for initial tenderness, beef flavor intensity, browning flavor and overall tenderness when compared with roasting at 160°C. Overall, slower cook times at lower temperatures resulted in improved palatability for the SM standard oven roast.

**Table 3:** Warner–Bratzler shear forces (WBSF) and palatability attributes^a^ evaluated by trained panellists of beef s*emimembranosus* and *longuissimus lumborum* roasts cooked at different searing time, searing temperature and roasting temperature.

Variable	STI, min				STE, °C		RTE, °C		SEM	*P*‐value		
	0	10	20	30	232	260	135	160		STI	STE	RTE
*Semimembranosus*												
WBSF, kg	7.91	7.65	6.90	7.65	7.51	7.54	7.28	7.77	0.263	0.147	0.931	0.129
Initial tenderness	5.85	5.94	5.79	5.75	5.84	5.83	5.99	5.67	0.106	0.796	0.942	0.015
Juiciness	4.81	4.84	4.94	4.82	4.90	4.80	4.95	4.75	0.083	0.810	0.342	0.066
Beef flavor intensity	4.90	4.55	4.60	4.71	4.69	4.69	4.80	4.59	0.080	0.073	0.948	0.025
Browning flavor intensity	3.14	2.91	2.87	2.81	2.91	2.96	3.04	2.83	0.070	0.064	0.626	0.013
Amount of connective tissue	6.51	6.61	6.49	6.36	6.42	6.56	6.57	6.41	0.083	0.542	0.208	0.152
Overall tenderness	5.81	6.04	5.82	5.74	5.82	5.88	6.02	5.69	0.096	0.412	0.628	0.008
*Longissimus lumborum*												
WBSF, kg	5.78	5.79	5.02	4.97	5.38	5.40	5.23	5.56	0.226	0.056	0.960	0.240
Initial tenderness	6.62	6.23	6.23	6.32	6.29	6.41	6.37	6.33	0.090	0.052	0.321	0.721
Juiciness	4.74	4.96	4.92	4.83	4.86	4.87	4.88	4.85	0.093	0.574	0.940	0.807
Beef flavor intensity	4.41	4.50	4.28	4.31	4.45	4.30	4.37	4.39	0.066	0.214	0.068	0.808
Browning flavor intensity	2.71^a^	2.77^a^	2.56^ab^	2.50^b^	2.77	2.50	2.69	2.58	0.060	0.048	0.001	0.158
Amount of connective tissue	7.28	7.15	7.13	7.16	7.19	7.17	7.18	7.18	0.046	0.214	0.694	0.942
Overall tenderness	6.38	5.95	6.15	6.18	6.13	6.20	6.16	6.18	0.113	0.179	0.591	0.897

STI, Searing time; STE, Searing temperature; RTE, Roasting temperature; SEM, Pooled standard error of least square means.

^a,b,c^Least squares means within a row lacking a common superscript letter differ (*P *<* *0.05).

a Sensory score were on an 8‐point scale: 1 = Extremely tough/dry/bland or none/abundant; 8 = Extremely tender/juicy/intense/none detected.

In contrast to the SM, roasting temperature had no effect on palatability attributes in the premium LL oven roasts (*P *>* *0.05). However, searing LL roasts for 0 and 10 min increased the browning flavor score compared to searing for 30 min; searing for 20 min resulted in intermediate values (*P *<* *0.05). Searing at a lower temperature (232 vs. 260°C) also resulted in higher browning flavor scores, although both means were in the same scale and rated as ‘very bland’(*P *=* *0.001). Finally, there was a tendency (*P *=* *0.052 and 0.056, respectively) for higher initial tenderness scores (more tender) and higher WBSF (less tender) in the 0 min sear samples compared to more extensive searing times. It is unknown if this was due to an experimental sampling error (shear force and panellist tenderness were not rated on the exact same samples) or whether it may be due to an inherent friability of the cooked samples that is detectable by panellists but not by WBSF.

### Variation in roast weights

As a whole, including the consumers’ desire for minimizing preparation and cooking times, the results indicated a reasonable roasting recommendation may be to oven sear for a maximum of 10 min at 232°C followed by roasting at 135°C. Conclusions drawn from the first experiment resulted in SM standard roasts of different weights were seared for 10 min in a 232°C oven, followed by dropping the oven temperature to 135°C and roasting to a final end point temperature of 68°C.

Cooking time per unit mass was found to be inversely related to roast weight, with larger roasts requiring less cook time per g than smaller roasts (Table 4). Roast weights ≥1 kg had proportionally lesser cooking losses, higher moisture losses on slicing and lower protein content than roasts weighing 0.5 kg. Panellists tended to rate 0.5 kg roasts higher for external browning (P = 0.08), and image analyses indicated roasts ≥1 kg had a more uniform internal color as fewer color bins were required to hold 75% of the pixels (*P *=* *0.001). As well, roasts ≥1 kg had significantly lower WBSF values compared to roasts weighing 0.5 kg.

**Table 4:** Cooking traits, postcooking proximate analysis, color parameters and Warner–Bratzler shear forces (WBSF) of beef s*emimembranosus* at different roasting weights.

Variable	Roasting weight, kg				SEM	*P*‐value
	0.5	1.0	2.0	2.5		
Cooking time, sec/g	8.03^a^	7.28^ab^	5.50^bc^	4.80^c^	0.750	0.017
Cooking loss, %	28.4^a^	25.0^b^	23.2^b^	24.0^b^	0.960	0.005
Moisture loss during slicing, %	1.24^c^	1.69^bc^	2.98^a^	2.63^ab^	0.400	0.014
Moisture, %	65.7	66.3	65.4	65.5	0.480	0.586
Fat, %	2.15	2.87	3.70	3.74	0.490	0.195
Protein, %	31.5^a^	30.0^b^	30.0^b^	29.9^b^	0.390	0.035
External Browning	3.11	2.27	1.89	1.60	0.430	0.087
Internal Doneness	5.47	5.83	6.29	6.04	0.300	0.253
Total Bins used	139.1	133.3	134.9	147.6	6.480	0.403
Bins holding 75% of pixel	8.17^a^	6.22^b^	5.58^b^	5.91^b^	0.390	0.001
WBSF, kg	8.46^a^	6.42^b^	5.72^b^	6.51^b^	0.530	0.013

^a,b,c^Least squares means within a row lacking a common superscript letter differ (*P *<* *0.05).

## Discussion

In general, modern consumers have less knowledge of meat selection, preparation, and cooking than those of generations past (BIC 2004). This limited knowledge, coupled with consumer time constraints, has resulted in home‐reduced preparation of traditional cuts, such as roasts (BIC 2004). However, consumers appreciate the roast experience available at food service and have a willingness to embrace more complex cooking instructions and longer cooking processes during their leisure time, when a premium experience can be guaranteed (BIC 2004).

Oven searing has been suggested as a means to perhaps enhance the quality attributes while minimizing the extra preparation and clean‐up associated with stove‐top searing (Joyce Parslow, Pers. Comm.). Based on our current results, the over‐riding effects of oven‐searing will be to increase the average cooking temperature and decrease the average cooking time, since there is a significant delay (average 60 min; data not shown) in the cooling of kitchen ovens from the elevated sear temperatures (232 or 260°C) to normal roasting temperatures (135 or 160°C). As a result roasts undergoing oven searing in this study, regardless of the connective tissue composition of the roast (SM or LL), had higher moisture loss and required less time to reach 68°C.

These differences in cooking time which occur through application of different cooking methods were expected to significantly affect many of the subsequent cooking and palatability attributes due to the time/temperature effect on denaturation of proteins (Jeremiah and Gibson 1999; Tornberg 2007; Aalhus et al. 2007). Although it has been reported (Kemp et al. 2003) that heat transfer capacities could be affected by the internal structure of different meat cuts/muscles (i.e., fat composition or connective tissue content), in the current study, SM and LL roasts showed similar cooking performance (i.e., searing for 0 min resulted in longer cooking times and less cooking losses than searing for 30 min).

Some researchers (Powell et al. 2003; Jeremiah and Gibson 1999; Ko et al. 2006) have investigated cooking protocols (i.e., braising, holding, etc.) within forced‐air convection ovens to fixed or variable end point temperatures. Jeremiah and Gibson (1999) evaluated different oven cookery protocols on five different beef roast cuts (inside round = *semimembranosus*; outside round = *biceps femoris*; eye of round = *semitendinosus*; rump = *rectus femoris*, and sirloin tip = *vastus lateralis*). With all five roast cuts, more cooking time was required to reach a final end point temperature (67.5°C) with constant dry heat roasting at 140°C, without a difference in cooking loss. The exception was the rump roast which had more cooking loss when cooked with an initial moist heat followed by a dry heat finish. The results of the current study agree with these findings. In other oven cooking protocols, Powell et al. (2003) compared beef ST roasts cooked in a conventional forced‐air convection oven (cooking at 163°C to a core end point of 65°C) versus multi‐stage cooking (preheating, holding 60 min at 55°C internal core, and finishing at 65°C). No differences related to cooking method were found in cooking yields. However, Ko et al. (2006) found greater cooking loss in a two‐step oven heating process (long low temperature and finishing with short high temperature) than conventional forced‐air convection oven in beef LL.

In the current study small SM standard oven roasts (0.5 kg) had higher cooking times and greater moisture losses. Due to the size of the roast the wall height of the roasting pans may have interfered with the circulation of heat reducing heat transfer compared to larger roasts. In addition, some researchers have indicated that forced‐air convection oven cooking is less efficient in transferring heat due to different air velocity distribution or heat fluxes in the oven cavity (Wahlby et al. 2005; Spence et al. 1984; Yancey et al. 1992). Cut shape has also been found to affect heat transfer rates and can have a great influence on eating quality. Aalhus et al. (2007) reported that of equal weight roasts of uniform cylindrical and square shape fabricated from the same muscle and cooked under similar conditions, cylindrical roasts had higher tenderness, juiciness and flavor scores. Similar results were reported by Bayne et al. (2011) who found that small rounds (0.85 kg) required almost four times longer to reach the end point at 93°C than large rounds (2.7 kg) and, as a result, cooking losses were higher.

Reduction of moisture content affects the relative composition of other chemical components. Thus, it was expected that fat levels would decrease (fat melts and drips out) and protein content increase as searing time or final internal temperature increased; but the results were inconsistent. Similarly, Booren et al. (2002) evaluated if holding time (from 0 to 120 min; dwell time) in ST roasts cooked to a designated end point temperature (60 or 66°C) in a water bath could affect the chemical components. At both end point temperatures, moisture and protein values for cooked meat samples decreased and increased; respectively, as cooking dwell time increased, although, levels of fat did not consistently decrease. In contrast, when conventional forced‐air convection oven was compared to a multi‐stage oven cooking procedure (preheating, holding 60 min at 55°C internal core, an finishing at 65°C) using beef ST roasts, no differences on fat or moisture content was found (Powell et al. 2003). Renk et al. (2000) found that internal temperature (68 vs. 79°C) and method of cookery (broiling vs. roasting) did not influence lipid concentrations of beef LL steaks; although, some researchers have indicated that cooking may contribute to fat losses and lipid oxidation (Rodriguez‐Estrada et al. 1985; Aalhus et al. 2007). During cooking, vitamins and minerals are lost, altering the nutritional value of the meat (Gerber et al. 1972). Potentially nutrient losses may be reduced by initial high‐temperature searing of the meat to create a surface barrier of denatured proteins (Aalhus et al. 2007).

The searing/roasting color results obtained in the current study could be explained by the slow heating rate which affects myoglobin denaturation occurring at approximately 63°C. During slow cooking, the core temperature will be similar to the surface resulting in uniform meat color (Aalhus et al. 2007). According to Bejerholm and Aaslyng (2004), a low heating temperature yields a more uniform appearance and less distinct layers of doneness. As well, the constant temperature will also ensure that the surface of the meat becomes brown due to a higher production of Maillard end products. Temperatures above 110°C and low water activity facilitate the Maillard reaction (Whitfield and Mottram 2000) and this would be increased if searing occurs through direct contact surfaces (e.g., grill or pan; (Barber and Broz 2008). Due to less efficient to heat transfer in small roasts (longer cooking times and increased cooking loss), the internal color was not uniform and the external browning score tended to increase.

Among Canadian roast beef eaters, relatively few prefer roast beef at either end of the doneness spectrum (6% Rare and 13% Well), with the majority closer to the middle (Medium‐rare = 33%, Medium = 28%, Medium‐well = 20%)(BIC 2004). Thus, slow cooking processes in the current study meets the consumer's preferences for a cooked appearance. Additionally, if end point temperature is manipulated, different degrees of doneness (Medium through Medium‐well) could be achieved which would have a uniform color when the rate of heating is slow.

Color evaluation of fresh meat through the use of colorimeters on nonuniform surfaces has limitations (averaging three point of reading on the sample without considering the whole area) particularly if the viewing port is small. Consequently, digital image analysis eliminates many restrictions of other instrumental methods and the whole view area can be analyzed, allowing a quantitative measure of color uniformity (Balaban 1995). In the current study, digital image analysis showed advantages as a tool for the determination of the uniformity of color. To the author's knowledge, no other publications addressing the color uniformity (pixel and bins) on cooked meat products exist. Some researchers have worked with this technique in fresh food such as mangoes, banana, fresh rabbit meat and no‐cooked fresh pork (O'Sullivan et al. 1997; Balaban 1995; Balaban et al. 2008). Objective measurement of the color such as L*, a*, b* values (i.e., Minolta) have been performance to evaluate internal color of cooked meat products (Lyon et al. 2000; Milligan et al. 1982; Hunt et al. 2009; Boles and Swan 2006; Yancey et al. 1992) where researchers have concluded that the end point temperature has a greater effect on cooked color (i.e., a* values decrease as end point temperature increase; less red color) than cooking method (Boles and Swan 2006; Yancey et al. 1992). Brownness increases due to increased myoglobin denaturation (Hunt et al. 2009). Unfortunately, color meters have small apertures and despite obtaining multiple readings on the sample, the uniformity of color across the whole area cannot be assessed. In addition small defects or other details of interest may be missed (Lu et al. 1977; Balaban 1995).

Consistently, cooking meat with slow rates of heating has been shown to improve tenderness through denaturation and shrinkage of collagen or preservation of enzyme‐activity (i.e., collagenase) at low cooking temperatures (Laakkonen et al. 2011; Seideman and Durland 2003; Tornberg 2007). Recently irreversible dissociation of actin and myosin has been shown to occur when cooking at low temperatures (improving tenderness) whereas denaturation of the actomyosin complex without dissociation (decreasing tenderness) occurs when cooking at higher temperatures (80°C) (King and Whyte 2009). Jeremiah and Gibson (1999) reported that *semitendinosus* roast prepared using high temperature moist heat were tougher, less juicy, less flavorful and less desirable than those prepared with low temperature dry/moist heat or high initial temperature and subsequently reduced. In contrast to the results of the current study, Jeremiah and Gibson (1999) did not find difference on palatability attributes between low temperature dry heat versus high temperature initially and subsequently reduced. In other oven cooking protocols, Powell et al. (2003) found shear forces were lower for the multi‐stage‐cooked roasts (3.3 kg) than for the conventionally cooked roasts (4.73 kg), because the percentage of total insoluble collagen fraction decreased in the multi‐stage cooking procedure (43.9 vs. 55.3%). However, Ko et al. (2006) found no differences in tenderness and juiciness between the two‐step oven heating processes (long low roasting temperature, and finishing with short high temperature) and conventional forced‐air convection oven roasting in beef LL, although the two‐step oven heating process had greater cooking loss.

With regard to roast size, small roasts had higher shear force that was attributable to longer cooking times and greater moisture loss which produced a hardening of muscle fibers (Laakkonen et al. 1970a; Draudt 2008; Tornberg 2007). However, Bayne et al. (2011) reported that tenderness (shear force and tenderness scores) of rib cuts was not related to size, whereas small round roasts (with long cooking times and high cooking losses) sheared 1.6 kg lower than large round roasts when oven‐cooked at either 93 or 149°C. Nevertheless, juiciness was affected by temperature × size interaction indicating that small roasts were less juicy than large roasts when heated at 93°C but were juicier than large roasts when heated at 149°C. Overall, roasting at lower temperatures for longer times resulted in substantial improvement of palatability traits than oven searing.

## Conclusions

Undoubtedly, cooking procedures and methods can affect cooking characteristics, chemical composition, internal cooked color and palatability attributes of meat. According to the present results, oven searing has limited positive effects on cooking, color and palatability traits. This is primarily because of the long time (47 to 72 min) for ovens to return to the desired roasting temperature following oven searing. However, any cooking procedure which extended the cook time, including no searing and low temperature roasting had more impact on most of palatability attributes. Roast weight was also an important contributor to palatability traits. These data suggest consumers may not achieve optimum palatability results when cooking small roasts and that surface to volume ratios are an important factor when fabricating small roasts (i.e., square vs. cylindrical shape).

Since consumers have preferences for “Medium” to “Medium‐Well” degrees of doneness based primarily on the visual presence of the red/pink myoglobin pigment. They associate this color as strong indicator of expected tenderness, juiciness and flavor, which can be manipulated through end point temperature. A more uniform color can be achieved when the rate of heating is slow.

Based on the results, oven searing provides limited or no added palatability benefits for the consumer other than reducing total cooking time. Hence we would recommend roasting at low temperatures (135°C) for both premium (LL) and standard (SM) oven roasts as it results in less moisture loss, better external browning, improved color uniformity and, for standard oven roasts with higher connective tissue content, increased tenderness with enhanced palatability traits.

## Conflict of Interest

None declared.

## References

[fsn3229-bib-0001] Aalhus, J. L. , L. L. Gibson , and I. L. Larsen . 2007 Evaluation of common beef cooking recommendations for optimal flavour, juiciness, tenderness and texture. Final report to the Canadian Beef Information Centre.

[fsn3229-bib-0002] Aalhus, J. L. , Gibson, L. L. , & Larsen, I. L . 2007 Evaluation of common beef cooking recommendations for optimal flavour, juiciness, tenderness and texture. Final report to the Canadian Beef Information Centre. Agriculture and Agri‐Food Canada, Lacombe Research Centre Pp. 27

[fsn3229-bib-0003] Aalhus, J. L. , L. E. Jeremiah , M. E. R. Dugan , I. Larsen , and L. L. Gibson . 2004 Establishment of consumer threshold for beef quality attributes. Can. J. Anim. Sci. 84:631–638.

[fsn3229-bib-0004] Aldai, N. , J. L. Aalhus , M. E. R. Dugan , W. M. Robertson , T. A. McAllister , L. J. Walter , et al. 2010 Comparison of wheat‐ versus corn‐based dried distillers’ grains with solubles on meat quality of feedlot cattle. Meat Sci. 84:569–577. 20374826 10.1016/j.meatsci.2009.10.014

[fsn3229-bib-0005] AMSA 1995 American Meat Science. Research guidelines for cookery, sensory evaluation and instrumental tenderness of fresh meat. Natl. Live Stock and Meat Board, Chicago, IL.

[fsn3229-bib-0006] Balaban, M. O. 2008 Quantifying nonhomogeneous colors in agricultural materials Part I: method development. J. Food Sci. 73:S431–S437. 19021817 10.1111/j.1750-3841.2008.00807.x

[fsn3229-bib-0007] Balaban, M. O. , J. Aparicio , M. Zotarelli , and C. Sims . 2008 Quantifying nonhomogeneous colors in agricultural materials. Part II: comparison of machine vision and sensory panel evaluations. J. Food Sci. 73:S438–S442. 19021818 10.1111/j.1750-3841.2008.00967.x

[fsn3229-bib-0008] Barber, N. , and C. Broz . 2011 The meat searing process: is sealing in juices fact or fiction? J. Culinary Sci. Technol. 9:99–105.

[fsn3229-bib-0009] Bayne, B. H. , B. H. Meyer , and J. W. Cole . 1969 Response of beef roasts differing in finish, location and size to two rates of heat application. J. Anim. Sci. 29:283–287.

[fsn3229-bib-0010] Bejerholm, C. , and M. D. Aaslyng . 2004 The influence of cooking technique and core temperature on results of a sensory analysis of pork ‐ Depending on the raw meat quality. Food Qual. Prefer. 15:19–30.

[fsn3229-bib-0011] BIC . 2006 Beef roasting study. The Canadian Beef Information Centre, Mississauga, ON P. 85

[fsn3229-bib-0012] Boles, J. A. , and J. E. Swan . 2002 Heating method and final temperature affect processing characteristics of beef semimembranosus muscle. Meat Sci. 62:107–112. 22061198 10.1016/s0309-1740(01)00234-0

[fsn3229-bib-0013] Booren, B. L. , J. L. Baumert , and R. W. Mandigo . 2005 Evaluation and composition of beef semitendinosus utilizing a novel cooking System. Nebraska Beef Cattle Reports. Pp. 93–95 available at: http://digitalcommons.unl.edu/animalscinbcr/153 (accessed December 14, 2011).

[fsn3229-bib-0014] Bouton, P. E. , and P. V. Harris . 1981 Changes in the tenderness of meat cooked at 50–65°C. J. Food Sci. 46:475–478.

[fsn3229-bib-0015] Bramblett, V. D. , R. L. Hostetler , G. E. Vail , and H. N. Draudt . 1959 Qualities of beef as affected by cooking at very low temperatures for long periods of time. Food Technol. 13:707–711.

[fsn3229-bib-0016] Canada Gazette . 1992 Part II: Livestock and Poultry Carcass Grading Regulations. Part III. Grade names and grade standards for beef carcasses. In http://laws.justice.gc.ca/eng/regulations/SOR-92-541/page-1.html (accessed November 17, 2011).

[fsn3229-bib-0017] Cheng, Q. , and D. W. Sun . 2008 Factors affecting the water holding capacity of red meat products: a review of recent research advances. Crit. Rev. Food Sci. Nutr. 48:137–159. 18274969 10.1080/10408390601177647

[fsn3229-bib-0018] Draudt, H. N. 1972 Changes in meat during cooking Pp. 243–259 *in* Proceedings 25th annual reciprocal meat conference. pp. 243–259. American Meat Science Association. Ames, Iowa, USA

[fsn3229-bib-0019] Gerber, N. , M. R. L. Scheeder , and C. Wenk . 2009 The influence of cooking and fat trimming on the actual nutrient intake from meat. Meat Sci. 81:148–154. 22063975 10.1016/j.meatsci.2008.07.012

[fsn3229-bib-0020] Hunt, M. C. , O. Sørheim , and E. Slinde . 1999 Color and heat denaturaion of myoglobin forms in ground beef. J. Food Sci. 64:847–851.

[fsn3229-bib-0021] Jeremiah, L. E. , and L. L. Gibson . 2003 Cooking influences on the palatability of roasts from the beef hip. Food Res. Int. 36:1–9.

[fsn3229-bib-0022] Kemp, R. M. , M. F. North , and S. R. Leath . 2009 Component heat capacities for lamb, beef and pork at elevated temperatures. J. Food Eng. 92:280–284.

[fsn3229-bib-0023] King, N. J. , and R. Whyte . 2006 Does it look cooked? A review of factors that influence meat color. J. Food Sci. 71:R31–R40.

[fsn3229-bib-0024] Ko, S. , S. H. Yoo , S. Lee , S. Cho , K. H. Kim , and R. Hwang . 2011 Effect of long low temperature short high temperature cooking cycle on physicochemical properties of beef. Food Sci. Technol. Res. 17:11–16.

[fsn3229-bib-0025] Laakkonen, E. , J. W. Sherbon , and G. H. Wellington . 1970a Low‐temperature, long‐time heating of bovine muscle 3. Collagenolytic activity. J. Food Sci. 35:181–183.

[fsn3229-bib-0026] Laakkonen, E. , G. H. Wellington , and J. W. Sherbon . 1970b Low temperature, long‐time heating of bovine muscle. 1. Changes in tenderness, water‐binding capacity, pH and amount of water‐soluble components. J. Food Sci. 35:175–177.

[fsn3229-bib-0027] Larmond, E. 1977 Laboratory methods for sensory evaluation of foods. Agriculture Canada, Ottawa, ON. Publ 1637.

[fsn3229-bib-0028] Lu, J. , J. Tan , P. Shatadal , and D. E. Gerrard . 2000 Evaluation of pork color by using computer vision. Meat Sci. 56:57–60. 22061771 10.1016/s0309-1740(00)00020-6

[fsn3229-bib-0029] Lyon, B. G. , B. E. Greene , and C. E. Davis . 1986 Color, doneness and soluble protein characteristics of dry roasted beef *semitendinosus* . J. Food Sci. 51:24–27.

[fsn3229-bib-0030] McDowell, M. D. , D. L. Harrison , C. Davey , and M. B. Stone . 1982 Differences between conventionally cooked top round roasts and semimembranosus muscle strips cooked in a model system. J. Food Sci. 47:1603–1607, 1612.

[fsn3229-bib-0031] Milligan, S. D. , M. F. Miller , C. N. Oats , and C. B. Ramsey . 1997 Calcium chloride injection and degree of doneness effects on the sensory characteristics of beef inside round roasts. J. Anim. Sci. 75:668–672. 9078482 10.2527/1997.753668x

[fsn3229-bib-0032] O'Sullivan, M. G. , D. V. Byrne , H. Martens , L. H. Gidskehaug , H. J. Andersen , and M. Martens . 2003 Evaluation of pork colour: prediction of visual sensory quality of meat from instrumental and computer vision methods of colour analysis. Meat Sci. 65:909–918. 22063455 10.1016/S0309-1740(02)00298-X

[fsn3229-bib-0033] Powell, T. H. , M. E. Dikeman , and M. C. Hunt . 2000 Tenderness and collagen composition of beef semitendinosus roasts cooked by conventional convective cooking and modeled, multi‐stage, convective cooking. Meat Sci. 55:421–425. 22061574 10.1016/s0309-1740(99)00171-0

[fsn3229-bib-0034] Renk, B. Z. , R. G. Kauffman , and D. M. Schaefer . 1985 Effect of temperature and method of cookery on the retention of intramuscular lipid in beef and pork. J. Anim. Sci. 61:876–881.

[fsn3229-bib-0035] Rodriguez‐Estrada, M. T. , G. Penazzi , M. F. Caboni , G. Bertacco , and G. Lercker . 1997 Effect of different cooking methods on some lipid and protein components of hamburgers. Meat Sci. 45:365–375. 22061474 10.1016/s0309-1740(96)00123-4

[fsn3229-bib-0036] SAS . 2003 SAS/STAT user's guide: statistics. SAS Institute Inc, Cary, NC.

[fsn3229-bib-0037] Seideman, S. C. , and P. R. Durland . 1984 The effect of cookery on muscle proteins and meat palatability: a review. J. Food Qual. 6:291–314.

[fsn3229-bib-0038] Spence, C. J. T. , N. A. Buchmann , and M. C. Jermy . 2007 Airflow in a domestic kitchen oven measured by particle image velocimetry. *16th Australasian Fluid Mechanics Conference Crown Plaza, Gold Coast, Australia 2–7 December 2007*, 1364–1368.

[fsn3229-bib-0039] Tornberg, E. 2005 Effects of heat on meat proteins ‐ Implications on structure and quality of meat products. Meat Sci. 70:493–508. 22063748 10.1016/j.meatsci.2004.11.021

[fsn3229-bib-0040] Wahlby, U. , C. Skjoldebrand , and E. Junker . 2000 Impact of impingement on cooking time and food quality. J. Food Eng. 43:179–187.

[fsn3229-bib-0041] Whitfield, F. B. , and D. S. Mottram . 1992 Volatiles from interactions of Maillard reactions and lipids. Crit. Rev. Food Sci. Nutr. 31:1–58. 1734915 10.1080/10408399209527560

[fsn3229-bib-0042] Yancey, J. W. S. , M. D. Wharton , and J. K. Apple . 2011 Cookery method and end‐point temperature can affect the Warner–Bratzler shear force, cooking loss, and internal cooked color of beef longissimus steaks. Meat Sci. 88:1–7. 21185659 10.1016/j.meatsci.2010.11.020

